# Associations of maternal lifestyle factors with inadequate pregnancy weight gain: findings from the baseline data of the LIMIT prospective cohort study

**DOI:** 10.1007/s00394-024-03473-0

**Published:** 2024-08-21

**Authors:** Dana El Masri, Mulubirhan Assefa Alemayohu, Federica Loperfido, Irene Bianco, Chiara Ferrara, Rosa Maria Cerbo, Stefano Ghirardello, Maria Cristina Monti, Beatrice Maccarini, Francesca Sottotetti, Elisa Civardi, Francesca Garofoli, Micol Angelini, Hellas Cena, Rachele De Giuseppe

**Affiliations:** 1 Laboratory of Dietetics and Clinical Nutrition, Department of Public Health, Experimental and Forensic Medicine, University of Pavia, Pavia, 27100 Italy; 2 Biostatistics and Clinical Epidemiology Unit, Department of Public Health, Experimental and Forensic Medicine, University of Pavia, Pavia, 27100 Italy; 3 Department of Epidemiology, School of Public Health, Mekelle University, Mekelle, Ethiopia; 4 Neonatal Unit and Neonatal Intensive Care Unit, Fondazione IRCCS Policlinico San Matteo, Pavia, 27100 Italy; 5 Clinical Nutrition Unit, Department of General Medicine, Istituti Clinici Scientifici Maugeri IRCCS, Pavia, 27100 Italy

**Keywords:** Lifestyle factors, Mediterranean diet, Physical activity, Gestational weight gain

## Abstract

**Background/objectives:**

Gestational Weight Gain (GWG) impacts maternal and fetal health; deviations from optimal ranges pose health risks. Maternal lifestyle before and during pregnancy strongly influences GWG. This study explores factors linked to inadequate GWG, focusing on Mediterranean Diet (MD) adherence and specific food consumption.

**Subjects/methods:**

178 pregnant women were enrolled at Fondazione IRCCS Policlinico San Matteo (Pavia) during pre-hospital care before birth meeting inclusion/exclusion criteria. Sociodemographic data, pre-pregnancy BMI, GWG, MD adherence, physical activity (PA) levels, and smoking habits were retrospectively collected. Validated questionnaires adapted for the target group, assessed MD adherence and PA level. Participants were classified into adequate (AGWG) and inadequate GWG groups following IOM guidelines.

**Results:**

Among 200 pregnant women (aged 30–36), 37.1% experienced low GWG and 24.1% excessive GWG. Our study revealed a significant association between inadequate GWG and educational level (*P* = 0.011); pre-pregnancy BMI (*P* = 0.005); MD adherence (*P* = 0.008), and daily average consumption of vegetables (*P* < 0.001). Our results also showed that a lower risk of EGWG vs. AGWG was associated with daily average consumption of vegetables (RRR = 0.279, *P* = 0.004), while a higher risk of EGWG vs. AGWG was associated with high daily meat product consumption (> 1.5 portions/day) (RRR = 7.83, *P* = 0.03). **Conclusion.** These findings emphasize the importance of promoting lifestyle changes before and during pregnancy to tackle the increasing incidence of inadequate GWG and improve the health outcomes of both mother and child.

## Introduction

Gestational weight gain (GWG) is fundamental during the prenatal period, as it plays a crucial role in the health of the expecting mother and the developing baby [1]. Inadequate GWG is referred to as any value that falls below or above the guidelines previously developed by the Institute of Medicine (IOM) and updated in 2009 based on pre-pregnancy body mass index (BMI) as 12.5–18 Kg for underweight, 11.5–16 Kg for normal weight, 7-11.5 Kg for overweight, and 5–9 Kg for obesity [1, 2]. Inadequate GWG is accounting nowadays for more than half of the pregnant women population in most of the countries [3, 4]. Failure to meet the optimal GWG exposes mothers and their infants to increased health risks that are widely associated with short- and long-term negative consequences. These adverse health consequences include mothers’ risk of developing gestational diabetes mellitus (GDM) and postpartum weight retention which may lead to obesity [1, 5], giving birth to a large- or small-for-gestational-age infant [6, 7], low or high-birth-weight infant (LBW, HBW) [7], childhood obesity [5], and many other health complications [8, 9]. Goldstein et al. (2017) have conducted a systematic review and meta-analysis of diverse international cohorts, on a sample of more than 1 million pregnant women, where 47% of the sample gained weight that exceeds IOM recommendations, indicating a significant association with a higher risk of large-for-gestational-age, macrosomia, and cesarean delivery. While 23% of the sample gained weight less than the recommended range, indicating a significant association with a higher risk of small-for-gestational-age and preterm birth [6].

Apart from maternal age, pre-pregnancy BMI, and the physiological changes of pregnancy, lifestyle factors before and during pregnancy were found to have a great impact on GWG [2].

Several studies have demonstrated that inadequate GWG is associated with pre-pregnancy BMI [1, 10], maternal smoking habits and physical activity (PA) level [1, 11], educational level [12], smoking habits [13], and adherence to a balanced diet [10].

The existing body of literature provides multiple pieces of evidence supporting the significance of lifestyle interventions targeting eating habits and PA levels [1, 14, 15], before and during pregnancy, in achieving and maintaining an optimal GWG, as well as minimizing adverse maternal and neonatal health effects [2]. Multiple systematic reviews and meta-analyses have demonstrated a favorable impact of lifestyle factors on GWG including PA level [16], educational level [17], smoking habits [16], and adherence to the MD [18]. These positive effects encompass a decreased risk of developing GDM [19], overweight [19], obesity [19], experiencing preterm delivery [20], and other metabolic problems [6, 19]. In particular, adherence to the MD was shown to be also associated with a reduction in the health risks that may affect newborns and children, such as fetal growth alterations, inappropriate birth weight, prematurity, gastroschisis, and other problems [19].

Optimizing GWG within IOM recommendations is a public health concern that needs to be addressed to maintain the overall well-being of two specific vulnerable groups, pregnant women, and their infants [21]. Despite this, there is a paucity of evidence in Italy about the magnitude of GWG adequacy and its associated factors, particularly in the Lombardy region. Therefore, the primary goal of this study was to investigate the association of inadequate GWG with maternal lifestyle factors using baseline data from the LIMIT study.

## Materials and methods

### Study design and period

A cross-sectional study was conducted using baseline data from the ongoing LIMIT (Lifestyle and Microbiome Interaction Early Adiposity Rebound in Children) prospective cohort project [22]. LIMIT aims at identifying the longitudinal interplay between infant gut microbiome, infant/maternal lifestyle, and environmental variables, in children with early adiposity rebound, which is a risk factor for the development of childhood obesity. This assessment will be conducted at different points in time (T_0_, at delivery; T_1_, 1 month; T_2_, 6 months; T_3_, 12 months; T_4_, 24 months; T_5_, 36 months after birth).

### Study participants and setting

In the ongoing LIMIT prospective cohort study, a total of 200 pregnant women were enrolled between October 2022 and June 2024 during the pre-hospital care before birth at the UOC Neonatology and Neonatal Intensive Care, Fondazione IRCCS Policlinico San Matteo of Pavia (Pavia, Italy), of which 178 pregnant women were eligible and selected to this specific study. The overall selection was performed based on the inclusion and exclusion criteria previously defined in the LIMIT study protocol [22] and the completeness of the data was added as an additional criterion for inclusion and exclusion. The study followed the Helsinki Declaration, and the participants signed a consent form.

### Variables, data source, and measurements

At the delivery, women were investigated for i). sociodemographic characteristics (such as age, and educational level); ii). pre-pregnancy anthropometric data; iii). Lifestyle behaviors during pregnancy (such as eating habits including adherence to the MD, level of Physical Activity, and smoking habits) by using previously validated questionnaires and structured interviews.

### Anthropometric measurements

Two trained dietitians collected maternal anthropometric data (GWG). Pre-gravid weight (Kg) was self-reported by the participants and a Harpenden stadiometer with a fixed vertical backboard and an adjustable headpiece was used to measure height in centimeters (cm). Participants were asked to stand barefooted on the platform of the stadiometer, in a Frankfort plane position for an accurate measurement of their height.

BMI was then calculated using the standard formula BMI = Weight (Kg)/Height (m^2^), enabling the classification of participants into the class of women with low pre-pregnancy weight status (BMI < 18.5 Kg/m^2^); women with normal pre-pregnancy weight status (18.5 ≤ BMI ≤ 24.9 Kg/m^2^), and women with overweight (25 ≤ BMI ≥ 29.9 Kg/m^2^) or obesity (BMI ≥ 30 Kg/m^2^) statuses [23]. The total weight gained during pregnancy was also self-reported by the participants.

### Adherence to the mediterranean diet

The maternal adherence to the MD was retrospectively investigated by two trained dietitians, using the MEDI-LITE score obtained from a previously validated questionnaire [24] and then adapted for the pregnant women target group. It evaluates the frequency of consumption of nine food groups.

- A score of 0 to 2 was assigned to indicate 2 for a high frequency, 1 for a moderate frequency, and 0 for a low frequency of consumption of five food groups: fruits, vegetables, cereals, legumes, and fish and fish products.
- For the groups of meat and meat products, and dairy products, a score of 2 was assigned for items consumed with low frequency, 1 for those consumed with moderate frequency, and 0 for those consumed with high frequency.
- For alcoholic consumption, a score of 2 was assigned to the middle category (1–2 units of alcohol/day), 1 for the lowest category (1 unit of alcohol/day), and 0 for the highest category (> 2 units of alcohol/day). In our analysis, due to incomplete data, we excluded alcohol consumption from the Mediterranean diet adherence score calculation.
- The last section is for olive oil, a score of 2 was given for regular use, 1 for frequent use, and 0 for occasional use.

The overall score, after excluding the alcohol consumption score, ranged from 0 to 16, suggesting the greatest adherence for the highest score obtained. It was classified into tertiles, low, medium, and high adherence to the Mediterranean diet.

### Physical activity level

A section of a previously developed and validated questionnaire on an Italian youth population [25] was adapted and administered to our adult participants by deleting physical activities in the school environment to investigate physical activity patterns. This adapted version was also pre-tested on a sample of 24 subjects and revised accordingly. All answers were structured to quantify the time spent weekly in physical activity [25], including the activities spent during free time and screen time [25]. The questionnaire was composed of 5 multiple-choice questions that followed a Likert scale format comprising 4 choices (“Always”, “Often”, “Sometimes”, and “Never”) corresponding to a score between 0 and 3, where the highest score suggested the healthiest habit [25].

### Smoking habit

Smoking habits were collected through interviews, and the number of smoked cigarette packs per year was documented. Participants were categorized as those who had never smoked, quit smoking before or during pregnancy, or started or kept smoking during pregnancy.

### Gestational weight gain (GWG)

Total GWG was calculated as the difference between the participant’s weight at delivery time and the pre-pregnancy weight. Based on that, women were classified into 3 categories, according to the IOM (U.S.) and the National Research Council (U.S.) Committee to Reexamine IOM Pregnancy Weight Guidelines [26], as follows:

i. Women with Adequate GWG (AGWG; women with normal pre-pregnancy BMI, gaining from 11.5 Kg to 16 Kg during pregnancy);
ii. Women with Excessive GWG (EGWG; women with pre-pregnancy BMI indicating overweight or obesity, gaining more than 11.5 Kg or 9 Kg during pregnancy, respectively);
iii. Women with Low GWG (LGWG; women with low pre-pregnancy weight status, gaining less than 12.5 Kg).

### Statistical analysis

A descriptive analysis was performed to explore the distribution of variables across the study subjects by outcome status (AGWG, EGWG, LGWG), and summarized in terms of mean and standard deviations for continuous variables. While frequency and percentage were used for categorical variables. Variables with a *p*-value of less than 0.2 (*p* < 0.2) in the univariate analysis were selected for the multinomial logistic regression model, aiming at identifying lifestyle factors associated with inadequate GWG. Education level, pre-pregnancy BMI, physical activity score, Mediterranean diet adherence score, daily vegetable consumption, and daily consumption of meat and meat products fulfilled the criteria and were included as potential predictors in our multinomial logistic regressions. A forest plot with confidence interval and adjusted relative risk ratio (RRR) was used to visualize the presence and strength of association of those factors with inadequate GWG (EGWG and LGWG) considering AGWG as the reference category.

## Results

**Fig. 1:**
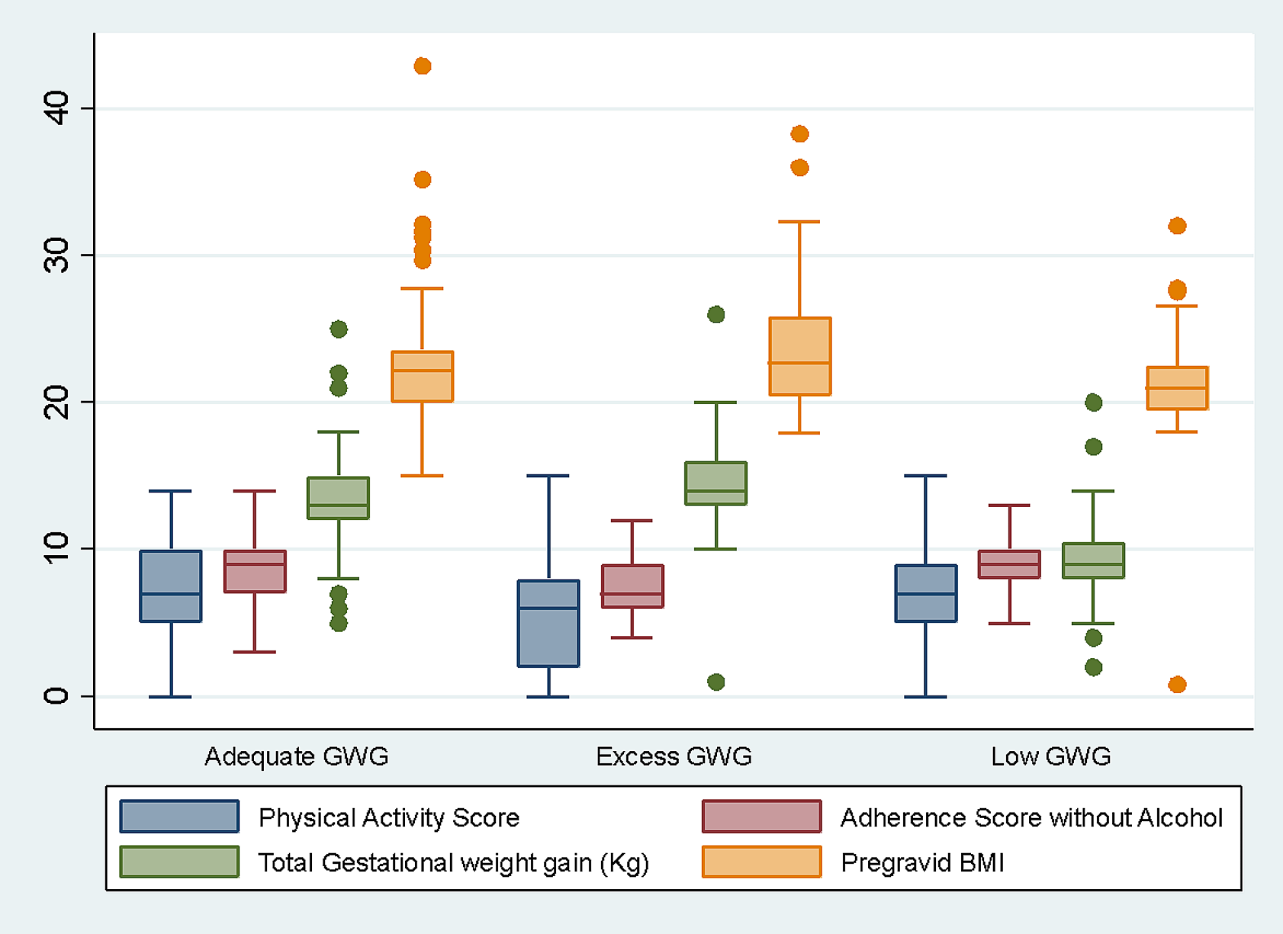
Distribution of physical activity score, Adherence to MD score, total gestational weight gain and pre-gravid BMI across Gestational Weight Gain Adequacy using LIMIT data *[0.055*,* 0.003*,* and 0.024 is the p-value for physical activity*,* pre-gravid BMI and adherence score*,* respectively*]

Table 1; Fig. 1 summarize the distribution of sociodemographic and lifestyle factors of the study participants based on the GWG category. More than half (61.2%) of the sample of study participants fell into the category of inadequate GWG, of which 60.5% and 39.4% were in the LGWG and EGWG categories, respectively.

Participants’ mean age was reported for each category as AGWG (mean age: 34 [32–37] years), EGWG (mean age: 33 [30–35] years), and LGWG (mean age: 32 [30–36] years), showing no statistically significant relationship with GWG. Concerning the educational levels, a significant association was detected (*P* = 0.0.011), where participants holding a university degree represented more than two-thirds of the AGWG (69%) and LGWG (79%) categories, compared to those with lower educational levels (31% of the AGWG; 21% of the LGWG). For the pre-pregnancy BMI variable, a statistical significance was observed (*P* = 0.0051), where the highest percentage of the participants in the EGWG category (63%), had a pre-pregnancy BMI indicating overweight or obesity. (Table 1).

Regarding the adherence to MD which showed a significant association (*P* = 0.008) with GWG, participants with highest and medium adherence scores represented almost half of the AGWG (52%) category and more than half of the LGWG (64%) category. On the other hand, those with the lowest adherence scores made up more than two-thirds of the EGWG (72%) category. (Table 2).

**Table 1:** Maternal sociodemographic and lifestyle factors related to gestational weight gain

	Gestational Weight Gain (GWG) Adequacy			*p*-value
	Adequate GWG	Excess GWG	Low GWG	
n	69 (38.8%)	43 (24.1%)	66 (37.1%)	
Age (year)	34.00 (32.00–37.00)	33.00 (30.00–35.00)	32.00 (30.00–36.00)	0.32
Mother’s Educational Level				0.011
Lower or High School	21 (31%)	21 (49%)	14 (21%)	
University degree	47 (69%)	22 (51%)	52 (79%)	
Did you smoke during pregnancy?				0.10
NO	66 (96%)	40 (93%)	66 (100%)	
YES	3 (4%)	3 (7%)	0 (0%)	
Pregravid BMI category				0.005
Underweight	9 (13%)	1 (2%)	6 (9%)	
Normal weight	46 (67%)	26 (60%)	55 (83%)	
Overweight	8 (12%)	10 (23%)	4 (6%)	
Obesity	6 (9%)	6 (14%)	1 (2%)	
Physical Activity				0.25
Lower Score	22 (32%)	20 (47%)	20 (30%)	
Medium Score	23 (33%)	15 (35%)	22 (33%)	
Higher Score	24 (35%)	8 (19%)	24 (36%)	

Legend. * Significance was set at *p*-value < 0.05

The individual items of the MEDI-Lite questionnaire were also considered; the results were summarized in Table 2 and categorized based on the 3 GWG groups, showing a highly significant association with the average daily consumption of vegetables (*P* < 0.001). Last, considering other lifestyle factors, participants with high and medium PA scores constituted the highest percentage of the AGWG (68%) and LGWG (69%) categories, while those with the lowest scores accounted for almost half of the EGWG category. Despite these differences, the association was not observed between PA and GWG (*P* = 0.25).

**Table 2:** Maternal diet consumption and adherence to the Mediterranean Diet by gestational weight gain (GWG) adequacy

	Gestational weight gain (GWG) adequacy			*p*-value
	Adequate GWG	Excess GWG	Low GWG	
MID Adherence Score category without alcohol				0.008
Lower Score	33 (48%)	31 (72%)	24 (36%)	
Medium Score	13 (19%)	3 (7%)	15 (23%)	
Higher Score	23 (33%)	9 (21%)	27 (41%)	
Daily Fruits Consumption				0.57
Inadequate	21 (30%)	13 (33%)	13 (20%)	
Average	40 (58%)	22 (56%)	44 (68%)	
Adequate	8 (12%)	4 (10%)	8 (12%)	
Daily Vegetables Consumption				< 0.001
Inadequate	7 (10%)	11 (28%)	8 (12%)	
Average	59 (87%)	23 (57%)	43 (65%)	
Adequate	2 (3%)	6 (15%)	15 (23%)	
Weekly Legumes Consumption				0.36
Inadequate	27 (40%)	15 (37%)	17 (26%)	
Average	29 (43%)	21 (51%)	39 (59%)	
Adequate	12 (18%)	5 (12%)	10 (15%)	
Daily CEREAL consumption				0.93
Inadequate	13 (19%)	8 (19%)	12 (18%)	
Average	39 (57%)	23 (53%)	33 (50%)	
Adequate	17 (25%)	12 (28%)	21 (32%)	
Weekly Fish Consumption				0.37
Inadequate	32 (49%)	17 (44%)	21 (33%)	
Average	31 (48%)	21 (54%)	39 (61%)	
Adequate	2 (3%)	1 (3%)	4 (6%)	
Daily Meat Consumption				0.11
Less than 1 portion	38 (56%)	19 (45%)	30 (46%)	
1 to 1.5 portion	28 (41%)	16 (38%)	31 (48%)	
>1.5 portion	2 (3%)	7 (17%)	4 (6%)	
Daily milk and milk product Consumption				0.25
Less than 1 portion	26 (38%)	15 (35%)	18 (28%)	
1 to 1.5 portion	35 (51%)	17 (40%)	36 (55%)	
>1.5 portion	8 (12%)	11 (26%)	11 (17%)	
Olive Oil Consumption				0.50
Occasional	7 (10%)	5 (12%)	4 (6%)	
Regular	50 (72%)	26 (60%)	45 (68%)	
Frequent	12 (17%)	12 (28%)	17 (26%)	

**Fig. 2:**
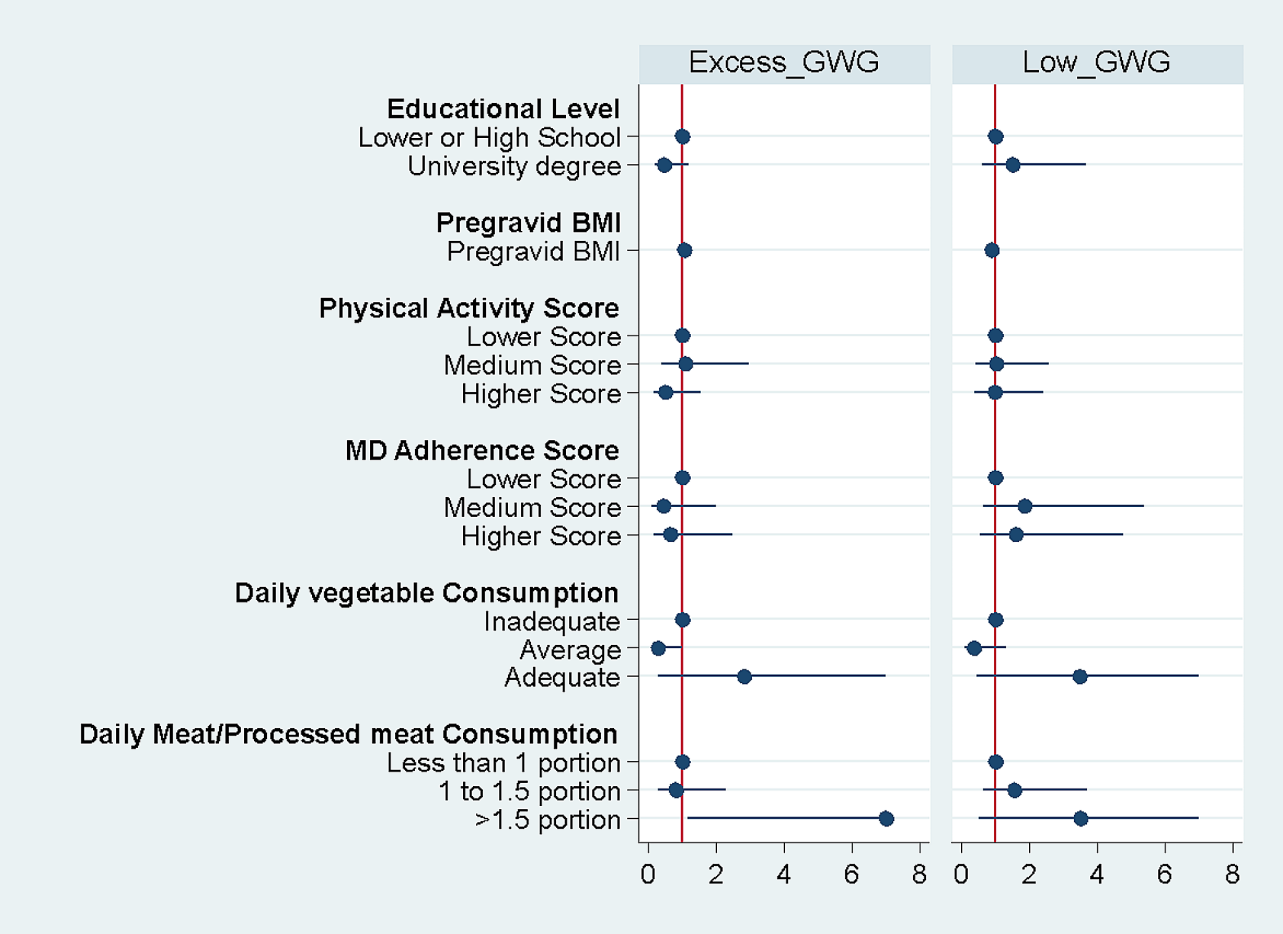
Forest plot showing the results of the multinomial regression model using baseline data from the LIMIT prospective cohort study. Predictor’s relative risk ratio and corresponding confidence intervals for Excess and Low GWG reported considering adequate GWG

Figure 2 represents a significant association between average daily consumption of 1 to 1.5 portions of vegetables and lower risks of EGWG when compared to the reference line of AGWG (RRR = 0.279, *P* = 0.04). A significant association was also represented between the daily consumption of > 1.5 portions of meat with higher risk of EGWG when compared to the reference line of AGWG (RRR = 7.83, *P* = 0.03). No significant evidence of any of the studied variables was detected in terms of LGWG in comparison with the reference line of AGWG (Fig. 2).

## Discussion

This study showed an alarming increase in the prevalence of inadequate GWG accounting for 61.2% out of 178 participants. Our findings indicate that the adequacy of GWG could be influenced by maternal factors such as pre-pregnancy BMI, educational level, adherence to the Mediterranean diet, and the average consumption of vegetables, as well as the excessive consumption of meat products. These findings may suggest the importance of promoting lifestyle interventions before and during pregnancy to ensure health and well-being for both, the mother and her infant.

No statistical significance supporting the evidence of the association between maternal age and inadequate GWG was observed. These results were inconsistent with a study conducted by Sun Y et al. [27] on a sample of 3172 pregnant women, where the authors found that an average age of 20–25 years old, is a protective factor for maintaining GWG within IOM guidelines against adverse health effects and malformations [27]. The inconsistency in findings between their results and the current study could be influenced by the huge difference in sample size of both studies. Furthermore, there was insufficient previous investigation about the effect of maternal age on GWG, which made it difficult to compare with other findings.

Current findings showed a significant association with maternal educational level, where 79% of the mothers who experienced an LGWG were holding a university degree. This result is not completely following the findings of a previous systematic review of observational studies that was conducted by O’Brien et al. [17], where pregnant women with low educational levels were less likely to maintain GWG within IOM recommendations. Further investigations must be performed on a larger sample targeting highly educated mothers to identify if their level of awareness about not gaining weight in excess during pregnancy, is negatively transformed into a GWG less than the recommendations.

Concerning pre-pregnancy BMI, our findings suggested a statistically significant association with GWG, as participants with pre-pregnancy overweight or obesity were more likely to experience EGWG, increasing the risk of adverse health consequences for the mother and her infant. This finding was consistent with previous studies, where women with a pre-pregnancy BMI value ≥ 25 Kg/m^2^, experienced EGWG and were at higher risk of developing GDM and/or pre-eclampsia, having preterm or cesarean birth, hemorrhage, infection and many other complications [1, 8]. EGWG has been reported with an increased likelihood to retain weight after delivery, which is usually followed by a higher possibility of developing postpartum obesity and suffering from other difficulties during future pregnancies, as well as giving birth to large-for-gestational-age infants, who will also be at higher risk to develop childhood overweight or obesity and its associated consequences [7, 9]. However, it was not possible to comment on the outcomes of our findings as this data was not yet collected.

In the present study, the adherence to the MD was significantly associated with GWG in comparison with IOM guidelines [1, 2], suggesting a potential benefit for the health of both, the mother and her infant. Participants with low adherence were more likely to experience EGWG. These results were in line with previous studies where lower GWG was associated with higher adherence to the MD before and during pregnancy [28–30]. In another systematic review, some of the selected studies highlighted the importance of MD during pregnancy to prevent excessive GWG, while others confirmed its association with a decreased risk of maternal and fetal complications [19]. This alignment with the existing literature confirms the importance of dietary interventions in the management of GWG and prevention of its adverse health consequences. Moreover, for a better understanding of this association, it is highly recommended to create or validate a questionnaire that is suitable for this population to detect adherence. Although our study did not measure the impact of MD adherence on newborns, the association with GWG suggests potential implications on the health of the infants and children. Previous studies also showed association between adherence to the MD and reduction of some pregnancy and childbirth complications as well as perinatal and childhood problems [19].

In terms of physical activity score, our findings imply no association with GWG, where score differences between GWG categories were relatively small, but higher among AGWG and LGWG indicating that more active pregnant women were less likely to gain excessive weight during pregnancy. This finding was in accordance with the systematic review results of O’Brien et al. [17] where PA was shown to be significantly inversely associated with GWG. It was also proposed by Teede et al. [14], that PA-based interventions are associated with reduced GWG as well as maternal and neonatal health risks. However, the highest mean value of PA score in our results was detected among the LGWG category, and this could also be linked to the high educational level of our participants. Perhaps, they tend to be more active due to their awareness of the importance of PA during pregnancy, leading to insufficient GWG. In 2019, O’Brien et al. [12] conducted a data meta-analysis about the impact of maternal education on GWG, indicating an increased risk of inadequate GWG among highly educated mothers following a mixed intervention of diet and PA-based interventions. Further investigations needed to be performed on a larger sample size, taking into consideration this relationship between educational level and physical activity and their association with GWG especially in terms of LGWG.

Maternal smoking habits were not associated with GWG. In contrast to our findings, a previously conducted systematic review by Zhou et al. [16], showed that smoking during pregnancy was associated with EGWG. However, our results may be influenced by the fact that only 6 participants from the whole sample were smokers, and those were equally distributed between AGWG and EGWG categories. Further examination is needed on a larger sample size.

Regarding the average daily consumption of vegetables that was shown in our study, to be associated with a lower risk of EGWG, our suggestion for future investigation is to identify if there is a link between high levels of maternal education and adequate consumption of vegetables on a larger sample size. This result could be considered following a previously conducted cohort study by Hirko et al. [31], where women with obesity who consumed more fruits and vegetables during pregnancy, were less likely to experience EGWG [31].

As for the excessive daily consumption of red meat, a significant association was detected with an increased risk of EGWG. A study conducted by Maugeri et al. [32], showed an increase in the trend of EGWG that was linked to the consumption of western dietary pattern, which means a high consumption of red meat and fast-food products [32].

Our study encompasses some limitations. The sample size is considered small, which may hinder the detection or be the reason for some associations and therefore affect the statistical power of the study. For this reason, we recommend future investigations incorporating larger sample sizes to better explore these associations. Unfortunately, we did not include other potential factors that could influence GWG such as “income”, since we did not collect this data, and “employment status” as there was a significant number of missing responses from our study participants. Additionally, pre-pregnancy weight and total GWG were self-reported by the participants and not measured by the trained dietitians, which increases the potential risk for bias by reducing the overall reliability of the data. Moreover, the lack of availability of a valid tool for assessing the adherence to the MD that is tailored to pregnant women, is a major limitation, as we had to adapt an existing questionnaire by removing the item of “alcohol consumption” and reducing the score, which affects its validity. Future research should focus on developing an assessment tool that is tailored to this specific population to enhance the robustness of the dietary assessment. However, this could also be considered as a strength of our study as we tried to make the measurement tool more relevant to our population to obtain meaningful data, as well as addressed a gap in the existing literature which is the absence of a valid questionnaire that measures adherence of the MD in pregnant women, taking into consideration the international guidelines in pregnancy which state the importance of avoiding alcohol within this critical period. Another key strength is the use of a section from the previously validated questionnaire by Turconi et al [25]. to assess PA levels among participants. Last but not least, our study provides a new region-specific data on GWG adequacy and its associated factors in the Lombardy region, offering insights to the policymakers and healthcare providers to develop new health interventions at the local level.

This study affirmed conclusions from previous research on the association between lifestyle factors including the adherence to the MD, before and during pregnancy, and the maintenance of GWG within IOM guidelines. An alarming increase in the rate of pregnant women with inadequate GWG was revealed, indicating a significant association with maternal educational level, pre-pregnancy BMI, adherence to the MD, average daily consumption of vegetables, and excessive consumption of meat products. The small sample size imposes a major limitation on our findings, however, the absence of a valid questionnaire that could assess MD adherence in pregnant women was detected as a gap in the literature and emphasized the importance of future research to fill it. Our results emphasize the importance of promoting lifestyle interventions during childbearing age as well as during pregnancy, as a crucial public health strategy to ensure health and well-being for two of the vulnerable groups, pregnant women, and infants.

## Data Availability

The data presented in this study are available on request from the corresponding author. The authors guarantee that the data that will be shared will comply with the consent given by the participants on the use of confidential data.
